# Fructose-induced metabolic reprogramming of cancer cells

**DOI:** 10.3389/fimmu.2024.1375461

**Published:** 2024-04-22

**Authors:** Kenneth K. Y. Ting

**Affiliations:** ^1^Department of Immunology, University of Toronto, Toronto, ON, Canada; ^2^Toronto General Hospital Research Institute, University Health Network, Toronto, ON, Canada

**Keywords:** fructose, cancer, metabolic reprogramming, tumor microenvironment (TME), ketohexokinase (KHK), GLUT5, metabolism, glycolysis

## Abstract

Excess dietary fructose consumption has been long proposed as a culprit for the world-wide increase of incidence in metabolic disorders and cancer within the past decades. Understanding that cancer cells can gradually accumulate metabolic mutations in the tumor microenvironment, where glucose is often depleted, this raises the possibility that fructose can be utilized by cancer cells as an alternative source of carbon. Indeed, recent research has increasingly identified various mechanisms that show how cancer cells can metabolize fructose to support their proliferating and migrating needs. In light of this growing interest, this review will summarize the recent advances in understanding how fructose can metabolically reprogram different types of cancer cells, as well as how these metabolic adaptations can positively support cancer cells development and malignancy.

## Introduction to metabolic reprogramming

Cancer cells undergo metabolic reprogramming, a process where they gradually acquire metabolic adaptations, to survive in a local tumor microenvironment (TME). These adaptations are critical in sustaining their high anabolic demands for uncontrolled proliferation, as well as conferring resistance against anti-tumor immunity. The first metabolic adaptation acquired by cancer cells was first observed by Otto Warburg in the 1920s (1, 2), where he found that cancer cells have adapted to oxidize glucose into lactate in greater amounts than other normal surrounding tissue despite residing in a normoxic environment. He later proposed that mitochondria dysfunction was the underlying cause behind the high aerobic glycolytic influx in cancer cells (3), which is now known as the Warburg effect. Since this discovery, many studies have now confirmed that cancer cells do undergo metabolic reprogramming, and further discovered that local cues in the TME, which is often influenced by lifestyle factors, can modulate the type of metabolic reprogramming undergone by cancer cells.

The daily consumption of fructose per person in United States has increased by 26% since the 1970s, highly correlating with the rise of incidence in obesity, metabolic diseases (ex. type 2 diabetes mellitus and non-alcoholic fatty liver disease (NAFLD)), neurological dysfunction and various types of cancer (4–7). This consequentially prompted the launch of many studies to determine if a TME enriched with high levels of fructose can reprogram cancer cells, enabling them to utilize fructose as a secondary source of fuel. As expected, an increasing number of studies have now shown that various types of cancer cells can indeed oxidize TME-derived fructose to support their proliferative and migrative needs, and these discoveries have been summarized in the following sections.

## Introduction to fructose metabolism

**Figure 1:**
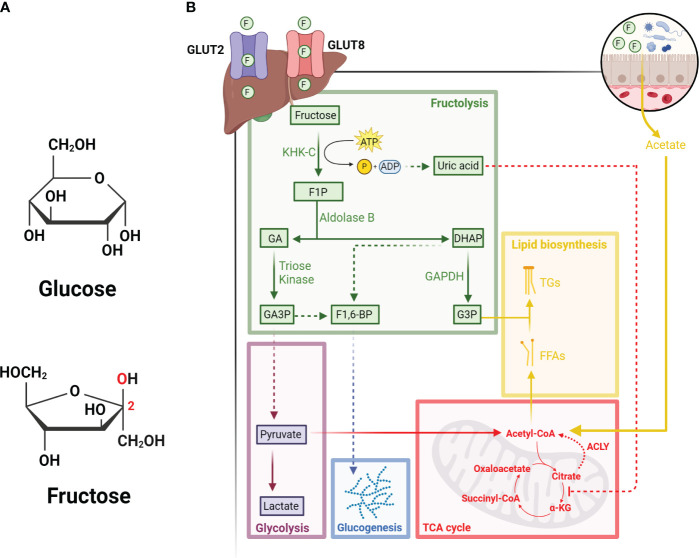
Hepatic metabolism of fructose. **(A)** Diagram illustrating the structural differences between glucose (top) and fructose (bottom). Red text indicates the carbonyl group located on carbon 2 of fructose. **(B)** Fructose enters hepatocytes from portal circulation through GLUT2 and GLUT8, followed by its phosphorylation by KHK-C into F1P. F1P is then converted into GA and DHAP by Aldolase B. GA can be phosphorylated by triose kinase into GA3P, which can either enter the glycolysis pathway or combine with DHAP to form F1-6-BP and enter the glucogenesis pathway. DHAP-derived from F1P can also be converted to G3P by GAPDH. The depletion of ATP due to rapid KHK-C activity leads to the accumulation of uric acid, which inhibits the activity of aconitase and increases the levels of citrate. Citrate can then be converted back to acetyl-CoA by ACLY and participate in the synthesis of free fatty acids (FFAs). Acetate derived from fructose metabolism in intestinal microbes also contributes to the lipogenic pools of acetyl-CoA. Finally, FFAs and G3P can be used to synthesize triglycerides (TGs), which can be stored as lipid droplets in the liver or secreted as very low-density lipoproteins into circulation. All figures are created with Biorender.com.

Although glucose and fructose share the same molecular formula (C_6_H_12_O_6_), their structural differences, such as the keto group in position 2 of the carbon chain in fructose, underlie their need to use different catabolic enzymes to facilitate their metabolism (**Figure 1A**). In general, the consumption of fructose (ex. in the form of sucrose or high fructose corn syrup) in hepatocytes is first phosphorylated to fructose-1-phosphate (F1P) by ketohexokinase (KHK) (8), which is a rate-limiting step of fructose metabolism. F1P is then converted into glyceraldehyde (GA) and dihydroxyacetone phosphate (DHAP) by Aldolase B (ALDOB). GA can then be phosphorylated into glyceraldehyde-3-phosphate (GA3P) by triose kinase and participate glycolysis as a substrate, or combine with DHAP to form fructose-1-6-bisphosphate (F1,6-BP) and enter the gluconeogenic pathway (9). Apart from this, F1P-derived DHAP can be converted to glycerol-3-phosphate (G3P) by Glyceraldehyde 3-phosphate dehydrogenase (GAPDH), in which G3P can then combine with free fatty acids to synthesize triglycerides (10) (**Figure 1B**).

Unlike glycolysis, where multiple enzymes (i.e., phosphofructokinase and glucokinase) can be regulated through feedback inhibition, fructose metabolism bypasses these checkpoints. For instance, KHK-mediated phosphorylation of fructose to F1P is not feedback inhibited, therefore excess dietary fructose metabolism can lead to rapid F1P generation, ATP depletion and consequentially uric acid production (11). On the other hand, the generation of F1P can also stimulate the activity of glycolytic enzymes, such as liver-type pyruvate kinase, thus increasing the rate of glycolysis and subsequently the TCA cycle (11). Since F1P-derived production of uric acid can induce mitochondrial oxidative stress and inhibit aconitase activity, high fructose metabolism through the TCA cycle can lead to the accumulation of citrate. As the levels of citrate accumulates, it can then be converted to acetyl-CoA by ATP Citrate Lyase (ACLY) and support *de novo* lipogenesis (12). However, recent research has also shown that microbial-derived acetate post fructose metabolism by gut-microbiota is the primary source that supplies the lipogenic pools of acetyl-CoA, a process independent of ACLY activities (13).

Apart from the liver, recent advances in understanding fructose metabolism also demonstrate that the small intestine in fact metabolizes fructose prior to the liver (14). Specifically, fructose is first imported in the gut intracellularly by GLUT5, which is expressed at the apical side of enterocyte luminal membrane (14, 15), as well as GLUT2 or GLUT5, which is expressed at the basolateral pole of enterocyte (16, 17). Upon entry into the enterocyte, fructose is rapidly metabolized. Any excess fructose that cannot be metabolized by enterocyte will be delivered from the portal circulation to the liver (18, 19) through GLUT2 (20–22) or GLUT8 expressed on hepatocytes (23).

## Colon-rectal cancer and intestinal cancer

The metabolism of colon-rectal cancer (CRC) is highly dependent on glycolysis to sustain their uncontrolled proliferation (24–27). In fact, the hyperactivity of glycolysis in CRC significantly depletes glucose in local environment, thereby priming CRC to use fructose as an alternative source to further fuel its proliferation. For instance, Shen et al. have reported that multiple human CRC cell lines (ex. HCT116 and HT29) upregulated GLUT5 expression in response to glucose depletion, which led to the increased uptake of fructose and its metabolism to fuel central carbon metabolism (28). Furthermore, the study also found that fructose-induced levels of GLUT5 proteins could bind to KHK and block its lysosomal degradation, further sustaining its metabolism of fructose (28). Similar findings were also reported by Włodarczyk et al where they inhibited the function of GLUT5 with N-[4-(methylsulfonyl)-2-nitrophenyl]-1,3-benzodioxol-5-amine (MSBNA) in a CRC cell line (HT-29), and this significantly reduced their viability. Taken together, these studies have demonstrated that GLUT5 plays a critical role in supporting the growth of CRC through mediating fructose metabolism (29).

As described previously, cancer cells undergo metabolic reprogramming to adapt and survive in local environment. This is particularly important for cancer cells that undergo metastases as they are required to migrate and proliferate in a new environment. For instance, CRC could metastasize in the liver, which is the current leading cause of cancer-related deaths (30, 31). Given that fructose is predominantly metabolized in liver, this raises the possibility that dietary fructose overconsumption can provide a secondary energy source for CRC to utilize upon its metastasis to the liver. Indeed, Bu et al. performed meta-analysis of clinical CRC liver metastases samples, as well as *in vivo* metastatic models, and found that glycolysis, glucogenesis, fructose metabolism and PPP were upregulated (32). Mechanistically, CRC-derived liver metastases upregulated ALDOB in a GATA6-dependent manner (32). This upregulation is critical for metabolizing fructose to fuel central carbon metabolism, thereby supporting their proliferation (32). Knocking down ALDOB or fructose-restriction inhibited the growth of CRC-derived liver metastasis (32).

Apart from CRC, Goncalves et al. have used mouse models to demonstrate that chronic fructose consumption facilitates intestinal tumor growth, a process independent of the effects of obesity and metabolic syndrome (33). Specifically, the authors found that intestinal tumors upregulate fructose metabolism, which is critical for fueling glycolysis due to F1P-induced ATP depletion (33). The enhancement of glycolysis subsequently led to increased fatty acid synthesis, a process vital for cancer cell growth due to its need for cellular membrane formation, energy generation, and intracellular signaling (34, 35). Overall, these studies have demonstrated that cancer cells that reside in organs that directly metabolize fructose, such as the liver and intestine, can undergo reprogramming and utilize fructose to support its metabolic needs.

## Melanoma and lung cancer

Although the effect of high fructose diet on CRC and intestinal cancer is well established, its effect on melanoma and lung cancer (LC) is conflicting, specifically in the context of anti-tumor immunity. Utilizing mice bearing B16 melanoma cells, Kuehm et al. found that the high levels of fructose in western diet led to the impairment of immune checkpoint blockade (ICB) therapeutic outcomes in these mice (36). Mechanistically, fructose activated cryoprotection in melanoma cells by inducing heme oxygenase-1 (HO-1) expression, allowing them to be resistant against immune-mediated killing during ICB therapy (36). Indeed, treating melanoma with inhibitor against HO-1 restored their susceptibility to ICB therapy both *in vitro* and *in vivo* (36). Similarly, in LC, GLUT5 expression was found to be induced, which led to the enhancement of fructose metabolism and increased synthesis of fatty acids (37). Notably, impairing GLUT5 expression in LC cell lines (ex. A549 and EKVX) significantly reduced its growth, while enforcing its expression exacerbated its malignancy (37). Similar findings were also reported by Weng et al, where they found that manipulating GLUT5 expression could modulate the malignancy of lung adenocarcinoma cells (ex. A549 and H1299), and that in comparison to glucose, these cells utilize fructose primarily for fatty acid accumulation and ATP production (38).

On the contrary, a recent study has shown that a high fructose diet is in fact beneficiary for anti-tumor immunity against melanoma and LC (39). Specifically, the authors in this study found that dietary fructose increased adipocyte-derived production of leptin, a hormone that is critical for T cell-mediated anti-tumor immunity (39). Indeed, B16-F10 melanoma-bearing mice with T cells that lack the expression of leptin receptor had increased tumor size post high fructose diet (39). Apart from this, the authors also selectively induced genetic deficiency of GLUT5 in adipocytes by utilizing *Adipoq-Cre : Slc2a5^fl/fl^* mice and found that upon their challenge with melanoma, these mice had lower serum leptin levels, lesser tumor-infiltrating CD8^+^ T cells and larger tumor size post high-fructose diet (39). Similar findings were reproduced in LLC murine lung cancer cells orthotopic tumor-bearing mice (39). Overall, these results have demonstrated that a high fructose diet may differentially affect the response of cancer cells against anti-tumor immunity.

## Breast cancer

In 1996, Zamora-León et al. first reported that breast cancer (BC) cell lines (ex. MCF-7 and MDA-468) selectively expressed GLUT5 (40), suggesting the possibility that BC can utilize fructose as a carbon fuel. Indeed, Fan et al. have later shown that BC cell lines (ex. MCF-7 and 4T1) incubated with fructose could proliferate at the same rate as incubating them with glucose, further demonstrating the metabolic plasticity of BC (41). Similar to other types of cancer cells, GLUT5 plays a critical role in supporting the growth of BC through mediating fructose metabolism as multiple studies have reported that impairing GLUT5 expression in BC cell lines (MCF-7 and 4T1) significantly reduced their proliferation rate (41, 42). Apart from proliferation, fructose metabolism has also been shown to enhance the migration ability of BC. For instance, Monzavi-Karbassi et al. reported that fructose could enhance the invasion of BC cell line (MDA-MB-468) (43). Specifically, the authors found that fructose altered cell-surface glycosylation of BC cells, which subsequently affected their binding to endothelial cells (43). Apart from this, fructose also changed the morphology and actin arrangement of these tumor cells, which correlated to an increase of their cell migration capacity (43). Similar findings were also reported by Jiang et al. where they found that fructose-induced 12-LOX signaling in 4T1 cells promoted their metastasis (44), as well as Fan et al. where they found that fructose increased metastasis of 4T1 cells *in vivo* (41). The detailed mechanism behind how fructose enhanced BC metastasis was then elucidated by Kim et al. where they found that fructose enhanced BC (MTV-TM-011) invasion in a KHK-A dependent manner (45). Mechanistically, KHK-A could bind to LRRC59 and KPNB1, transporting KHK-A into the nucleus where it could phosphorylate YWHAH, and promote SLUG recruitment to CDH1 promoter and reduced the expression of E-Cadherin (45). Taken together, these studies have collectively illustrated that fructolytic enzymes can support the growth and migration of BC through their classical role in mediating fructose metabolism, as well as performing moonlighting functions.

## Acute myeloid leukemia, pancreatic cancer, glioma

Abnormally high fructose levels have been previously reported in the bone marrow of acute myeloid leukemia (AML) patients (46), which raised the possibility that AML cells can also utilize fructose to fuel their growth. Indeed, Jeong et al. have reported that GLUT5 expression in a variety of AML cell lines (ex. MOLM13) is critical for regulating their fructose metabolism, which directly fuels the *de novo* serine synthesis pathway (SSP) (47). Specifically, the authors found that the increased activity of SSP in AML cells directly contributed to the increased production of α-ketoglutarate from glutamine, thus directly supporting TCA cycle anaplerosis (47). Knocking down Phosphoglycerate Dehydrogenase (PHGDH), a rate-limiting enzyme of SSP, reduced AML burden load in the presence of high fructose (47). Finally, the authors also found that AML cells unexpectedly utilized hexokinase (HK), rather than KHK, to metabolize fructose (47). Similar findings were also reported by Chen et al, where they have shown that various AML cell lines (ex. U937) upregulated GLUT5 and increased fructose metabolism under glucose-depleted condition to sustain their proliferation and survival (46). Metabolically, the enhanced fructose metabolism increased glycolytic flux in AML cells, as well as exacerbated their leukemic phenotypes (proliferation and migration) (46). Finally, inhibiting fructose metabolism in AML cells with *in vitro* and *in vivo* models with 2,5-anhydro-D-mannitol (2,5-AM), a fructose analog with high affinity to GLUT5, demonstrated therapeutic potential (46). Overall, these studies have shown that AML cells can oxidize fructose to support its growth through fueling its central carbon metabolism.

Like other types of cancer cells, fructose is also critical for supporting the growth of pancreatic cancer (PC). For instance, Carreño et al. showed that GLUT5 expression was upregulated in both human PC cell lines (ex. LNCaP and PC3) and malignant human prostate tissues, and that the enhancement of fructose metabolism is important for the growth of PC (48). Mechanistically, Liu et al. have shown that the metabolism of fructose in PC cell lines (ex. Panc-1 and MiaPaCa-2) was primarily used to support nucleic acid synthesis via the transketolase-regulated, non-oxidative branch of PPP (49). Specifically, the authors performed^13^C-labelled glucose and fructose tracing in PC cells and found that fructose was primarily oxidized to synthesize nucleic acids while glucose was oxidized to support glycolysis, TCA cycle and fatty acid synthesis. Overall, these studies have demonstrated that while fructose is important for the growth of PC, these cells have the ability to utilize glucose and fructose differentially to support its proliferation needs.

Similarly in glioma, Su et al. have reported that GLUT5 was overexpressed in both glioma cell lines (ex. LN22 and, U87) and glioma tissues, as well as demonstrating that glioma cell lines could utilize fructose to proliferate (50). In addition to this, the authors also found that inhibiting GLUT5 expression impaired glioma cells proliferation *in vitro* and the expression of GLUT5 was positively associated with poor survival of glioma patients (50). Apart from GLUT5, Gao et al. have also reported that KHK was overexpressed in glioma cells (ex. LN229 and U87), and that silencing its expression significantly reduced their proliferation and migration capacity (51). Furthermore, the authors have also discovered that fructose could enhance the stability of both mRNA and protein levels of KHK but the underlying mechanism behind its stabilization is not well understood (51). Taken together, both of these studies have confirmed that glioma can metabolize fructose to support its growth and migration by upregulating the expression of fructolytic enzymes, such as GLUT5 and KHK.

## Hepatocellular carcinoma

Finally, in contrast to other types of cancers, multiple studies have shown that fructose metabolism was in fact reduced in hepatocellular carcinoma (HCC) in comparison to normal hepatocytes (52, 53). For instance, Li et al. have reported that HCC utilized KHK-A, one of the two isoforms of KHK (with the other being KHK-C), to metabolize fructose. This finding was unexpected as KHK-C is primarily expressed in liver and it has a higher affinity for fructose than KHK-A (54). However, as illustrated by Li et al, HCC induced a c-Myc-dependent aberrant splicing change during the expression of KHK, which led to the production of KHK-A rather than KHK-C, and thus reducing the activity of fructose metabolism in HCC (52). Notably, the authors then found that this switch to KHK-A enabled it to phosphorylate phosphoribosyl pyrophosphate synthetase 1 and support nucleic acid synthesis through the PPP. Similar findings were also reported by Tee et al, where they have confirmed that KHK expression was downregulated in both HCC mouse models and human tissues, as well as demonstrating that the uptake of fructose in HCC cell lines (Huh7 and Hep3B cells) was reduced (53). More importantly, the authors have overexpressed KHK in HCC cell lines and found it significantly reduced their activity of glycolysis and TCA cycle, thus demonstrating that the suppression of KHK is a metabolic adaptation acquired by HCC to support their upregulation of glycolysis. The importance of glycolysis in facilitating HCC development under high fructose conditions has also been reported by Syamprasad et al, where they found that the expression of AKR1B1, the master regulator of polyol pathway that reroutes glucose to the generate sorbitol and fructose, was upregulated in HCC (55). Furthermore, the authors also found that inhibiting the function of AKR1B1 suppressed glycolytic reprogramming in HCC cell lines (PLC/PRF-5), while overexpressing it increased glycolysis. Finally, the authors discovered that inhibiting AKR1B1 reduced the pathologies induced by a high fructose diet and diethylnitrosamine administration, thus supporting the role that AKR1B1 plays in mediating the glycolytic reprogramming of HCC.

Apart from this, recent research has also shown that HCC can utilize the acetate secreted by gut microbes post high fructose metabolism (56). As previously described, microbial-derived acetate post fructose metabolism is critical for fueling the lipogenic pools of acetyl-CoA in hepatocytes (**Figure 1B**), and now a recent study performed from Zhou et al. have taken a step further and showed that microbial-derived acetate could also fuel *de novo* glutamine synthesis in HCC (56). This consequentially led to the enhancement of O-linked N-acetylglucosamine (O-GlcNAc) modification (O-GlcNAcylation), a post-translational modification of proteins that is critically dependent on the levels of glutamine. Specifically, the authors found that microbial-derived acetate enriched O-GlcNAcylation of CAPNS1 and eEF1A1, which are critical for cell proliferation and protein translational output, thus promoting the growth and metastasis of HCC. Taken together, this study has demonstrated that HCC can utilize microbial-derived acetate as a secondary source of carbon to meet its metabolic demands.

## Conclusion and future perspectives

**Figure 2:**
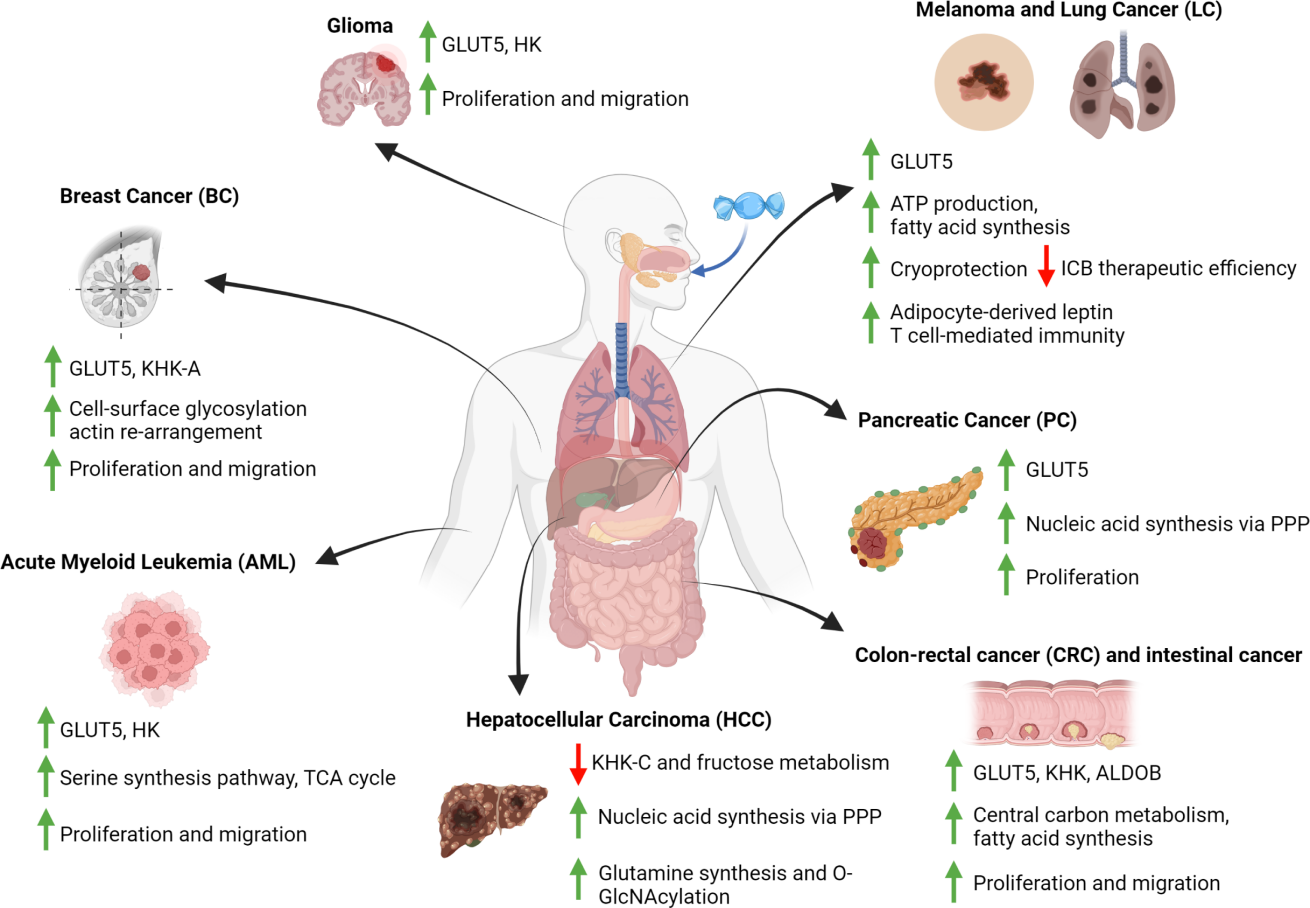
Fructose-induced metabolic reprogramming in cancer cells. Diagram that summarizes the metabolic consequences of fructose metabolism in various types of cancer cells. These consequences include changes to the expression of fructolytic enzymes, the fluxes of various metabolic pathways and the positive impacts to cancer cells development. All figures are created with Biorender.com.

Based on the studies reviewed above, several conclusions can be drawn on how fructose contributes to the metabolic reprogramming of cancer cells (**Figure 2**). Firstly, fructose can be utilized as an alternative carbon source to support central carbon metabolism, which is critical for *de novo* lipid and nucleic acid synthesis, and thus sustaining cancer cell proliferation. However, this seemingly only happens in the TME where glucose is depleted and fructose is in abundance, thereby priming cancer cells to use fructose as an alternative carbon source. Secondly, different types of cancer cells can utilize fructose or its derived metabolites differentially to support their metabolic needs. For instance, fructose is primarily used in PC to support nucleic acid synthesis through the non-oxidative branch PPP rather than fueling fatty acid synthesis. On the other hand, HCC reduced its intrinsic fructose metabolism and utilized acetate derived from fructose metabolism of gut microbes to meet its metabolic demands. Thirdly, the upregulation of fructose metabolism in cancer cells is always dependent on an increased activity (i.e., increased expression) of fructose metabolic enzymes. Finally, inhibiting the metabolism of fructose in cancer cells that were fructose-adapted significantly reduced their malignancy. This demonstrates that cancer cells do respond to high fructose in the TME, and their adaptation to fructose is critical for their survival.

Although the studies listed above elucidated how fructose supports the growth of various types of cancer cells, it remains unclear how the expression of fructose metabolic enzymes is regulated in response to fructose in the TME, including transcriptional and epigenetic mechanisms. A new study conducted by Huang et al. have shown that STAT6 is implicated in regulating the transcription in GLUT5 in a PC cell line (ex. DU145) (57), yet this remains to be characterized in other types of cancers. Secondly, how fructolytic enzymes can contribute to the pro-survival benefits of cancer cells, independent of their classical roles in metabolism, is only beginning to be appreciated, such as the secondary roles of KHK-A observed in BC (45) and HCC (38). Finally, it remains largely unclear how fructose-induced metabolic rewiring of cancer cells can modulate its interaction with immune cells. This is significant because a high fructose diet has always been linked to the induction of a chronic inflammatory environment (10), which is a critical factor that can modulate the development of cancer cells and anti-tumor responses (58). Therefore, future research should be warranted to investigate the consequences of excess dietary fructose metabolism, specifically how fructose diet-induced metabolites and inflammatory mediators can shape the TME and thus the crosstalk between tumor infiltrating immune cells and cancer cells.

## Author contributions

KT: Writing – original draft, Writing – review & editing.
